# A_2A_ Adenosine Receptor Antagonists and Their Efficacy in Rat Models of Parkinson’s Disease

**DOI:** 10.3390/cells14050338

**Published:** 2025-02-26

**Authors:** Andrea Spinaci, Michela Buccioni, Diego Dal Ben, Beatrice Francucci, Karl-Norbert Klotz, Gabriella Marucci, Nicola Simola, Micaela Morelli, Annalisa Pinna, Rosaria Volpini, Catia Lambertucci

**Affiliations:** 1 Medicinal Chemistry Unit, School of Pharmacy, University of Camerino, Via Madonna delle Carceri, I-62032 Camerino, Italy; andrea.spinaci@unicam.it (A.S.); michela.buccioni@unicam.it (M.B.); diego.dalben@unicam.it (D.D.B.); beatrice.francucci@unicam.it (B.F.); gabriella.marucci@unicam.it (G.M.); catia.lambertucci@unicam.it (C.L.); 2 Institut für Pharmakologie und Toxikologie, Universität Würzburg, Versbacher Str. 9, D-97078 Würzburg, Germany; karl-norbert.klotz@uni-wuerzburg.de; 3 Department of Biomedical Sciences, Section of Neuroscience, University of Cagliari, Cittadella Universitaria, SP 8, Km 0.700, I-09042 Monserrato, Italy; nicola.simola@unica.it (N.S.); morellimicaela@gmail.com (M.M.); 4 Neuroscience Institute, National Research Council of Italy (CNR), Cagliari Cittadella Universitaria, SP 8, Km 0.700, I-09042 Monserrato, Italy

**Keywords:** adenosine receptor ligands, adenosine receptor antagonists, synthesis of purine derivatives, binding and functional assays, *in vivo* models of Parkinson’s disease, medicinal chemistry

## Abstract

Parkinson’s disease (PD) represents a growing challenge to global health, as it involves millions of people. The high grade of disability is due to the loss of dopaminergic neuron activity, and levodopa is the gold-standard therapy used to restore dopamine in the dopamine-denervated regions. Another therapeutic approach is the use of A_2A_ adenosine receptor antagonists and, among them, istradefylline is the only one currently approved for therapy in association with levodopa. In this work, we synthesized A_2A_ adenosine receptor antagonists represented by 9-ethyl-2,8-disubstituted adenine derivatives, which were tested at human adenosine receptors in binding and functional assays. These compounds showed A_2A_ adenosine receptor-binding affinities in the low nanomolar range and **1**, **4**, and **5** exhibited good potency in the functional assays. Hence, they were evaluated in *in vivo* rat models of PD, where they were demonstrated to revert haloperidol-induced catalepsy and potentiate levodopa-induced contralateral rotations in 6-hydroxydopamine-lesioned rats. The most potent derivative, **4**, was then evaluated in the tacrine model, where it reduced the tremulous jaw movements, therefore demonstrating an action on parkinsonian tremor. These data revealed 8-ethoxy-2-phenethoxy-9-ethyladenine (**4**) as an A_2A_ adenosine receptor antagonist endowed with antiparkinsonian effects and as a good candidate to treat the disease.

## 1. Introduction

Parkinson’s disease (PD) is a neurodegenerative disorder with a marked increasing trend in patients worldwide; in fact, it is considered the second most common neurodegenerative disease, being a growing challenge to global health [1]. Several factors could be related to the pathogenesis of PD, producing a loss of function of the dopaminergic system [2,3,4]. The most used therapies try to restore the function of this system in the brain, but they produce undesirable side effects by becoming problematic with continued treatment [5]. In particular, the most efficacious approach is the restoration of dopamine levels by the administration of the precursor levodopa (L-dopa) [5].

Another strategy is the modulation of the A_2A_ adenosine receptor (A_2A_AR), which negatively influences the activity of dopaminergic D2 receptors [6,7,8].

The A_2A_AR is one of the four AR subtypes called A_1_, A_2A_, A_2B_, and A_3_, which are G-protein-coupled receptors belonging to family A of rhodopsin receptors [9] and are widely localized in all the cells with different concentrations [10].

In the striatum, the A_2A_/D_2_ receptor dimerization means that the antagonism of the A_2A_AR positively modulates the D_2_ receptor activation by dopamine, ameliorating the symptoms of patients affected by PD [11,12,13].

**Figure 1:**
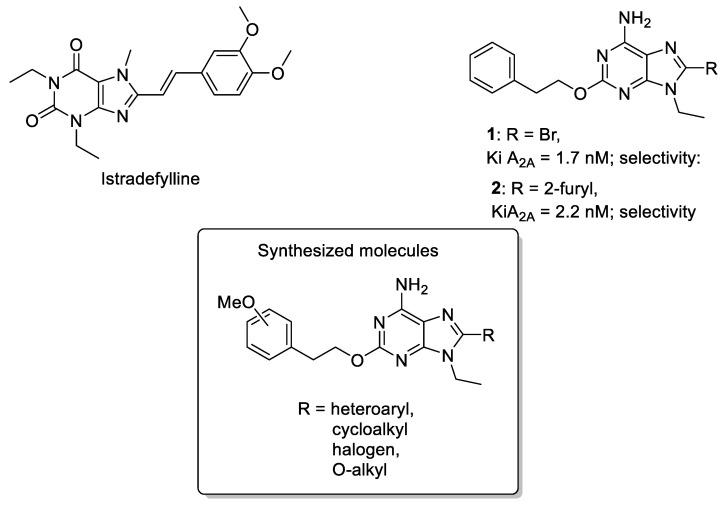
Known A_2A_AR antagonists and synthesized ligands.

In recent decades, great efforts have been employed toward the identification of new tools able to block A_2A_ARs, but, to date, only the xanthine derivative istradefylline (Figure 1) is commercially available for the treatment of PD in Japan and the USA under the trade names of Nouriast^®^ and Nourians^®^, respectively [14,15]. In Europe, istradefylline underwent clinical trials, but the EMA, after re-examination, did not recommend the marketing authorization for Nouryant^®^ (the European name of the medical specialty containing istradefylline) [16].

The structures of A_2A_AR antagonists are characterized by different heterocyclic scaffolds [17]. Among adenine derivatives, the 9-ethyl-8-ethoxyadenine (ANR 94), showing good affinity and selectivity at the human A_2A_AR (*K*_i_ = 46 nM), was demonstrated to counteract parkinsonian symptoms in rat models of PD [18]. On the other hand, the introduction of a 2-phenylethyloxy group at the 2-position of 9-ethyladenine led to high A_2A_AR affinity ligands, especially when a bromine atom or a 2-furyl ring were introduced at the 8-position of the purine moiety [19,20]. In fact, the 8-bromo-9-ethyl-2-phenetoxyadenine (**1**) and 9-ethyl-8-furyl-2-phenetoxyadenine (**2**; Figure 1) showed *K*_i_ values at the human A_2A_AR of 1.7 nM and 2.2 nM, respectively.

Hence, with the aim at finding A_2A_AR antagonists endowed with high affinity, a series of 2-phenethoxyadenine derivatives substituted at the 8-position with different groups have been synthesized. Some of them are characterized by the presence of a methoxy group at the phenyl ring of the 2-chain (Figure 1).

**Scheme 1:**
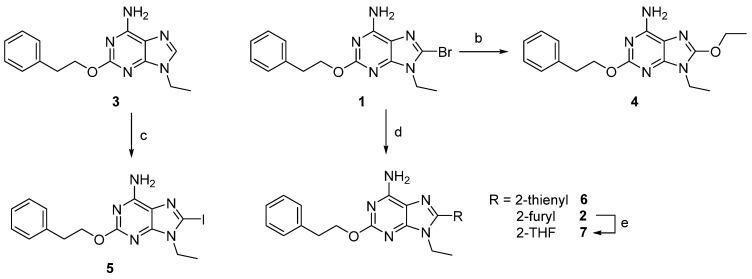
Synthesis of 9-ethyl-2-phenylethyloxy-8-substituted adenines. Reagents and conditions: a. NBS, DMF, r.t., 45′; b. EtOH, NaOH, 95 °C, 7 h; c. (i) LDA, THF dry, −70 °C, 1 h; (ii) I_2_, THF, −70 °C-r.t. 2.5 h; d. ArSnBu_3_, (Ph_3_P)_2_PdCl_2_, THF dry, 60 °C, 2.5 h; e. H_2_ 13 atm, PdO, HCl, *i*PrOH, 65 °C, 7 h.

**Scheme 2:**
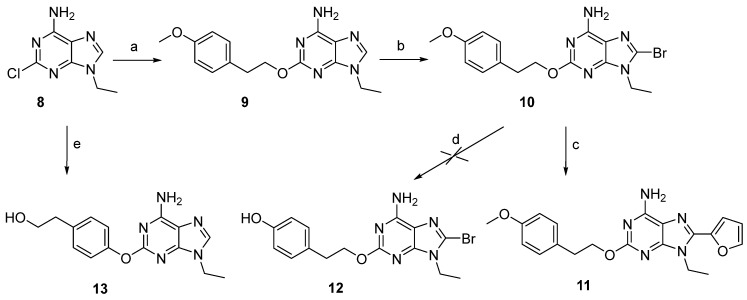
Synthesis of 9-ethyl-2-(4-methoxyphenethoxy)-8-substituted adenines. Reagents and conditions: a. (4-O-CH_3_)-Ph(CH_2_)_2_OH, NaOH, 85 °C, 4 h; b. NBS, DMF, r.t., 10 min; c. 2-FurylSnBu_3_, (Ph_3_P)_2_PdCl_2_, THF, 60 °C, 5 h; d. HBr, 100 °C; e. (4-OH)-Ph(CH_2_)_2_OH, NaOH, MeCN dry, reflux, 24 h.

The synthesis and ^1^H-NMR spectra of most compounds here reported has been already published in two patents [21,22], while their binding and functional data and anti-Parkinson activity has never been reported. For these reasons, here we reported only a general synthesis description and experimental procedures to synthesize compounds **5**, **10,** and **13** (Scheme 1 and Scheme 2), not reported in the cited patents.

The compounds were tested in binding studies at ARs to evaluate their A_2A_AR affinity and selectivity versus the other subtypes. Some of those selected were also tested in functional assays to verify their A_2A_AR antagonistic behavior and their efficacy as antiparkinsonian agents through the use of specific experimental *in vivo* rat models of PD.

Catalepsy induced by the dopamine receptor antagonist haloperidol is the most common pharmacological model of PD used to screen the antiparkinsonian properties of drugs.

Furthermore, in the model of rodents characterized by a unilateral lesion of dopaminergic nigrostriatal neurons with the neurotoxin 6-hydroxydopamine (6-OHDA), the antiparkinsonian activity of a specific drug can be measured by its ability to increase the rotational behavior induced by dopamine receptor agonists [18,23,24].

Moreover, a specific experimental model of parkinsonian-like tremors characterized by tremulous jaw movements (TJMs) induced by the acetylcholinesterase inhibitor tacrine has been validated for evaluating the anti-tremorigenic effects of antiparkinsonian drugs [25]. TJMs induced by cholinomimetic drugs are similar to human parkinsonian tremors in regard to many electromyographic and pharmacological characteristics [26]. The predictive validity of this model has been confirmed by the fact that TJMs can be attenuated by clinically effective antiparkinsonian drugs [27] and by the acute administration of A_2A_AR antagonists [23,24,28,29].

Therefore, the *in vivo* antiparkinsonian properties of the three synthesized A_2A_ antagonists, **1**, **4,** and **5**, were evaluated with these two different validated rodent PD models: (1) counteraction of catalepsy induced by the haloperidol, and (2) potentiation of contralateral rotations induced by L-dopa in unilaterally 6-OHDA-lesioned rats [18,23]. Finally, the compound that showed the highest antiparkinsonian efficacy in those models was tested using the TJMs model [23].

## 2. Materials and Methods

### 2.1. Chemistry

#### 2.1.1. General Methods

Melting points were determined with a Büchi apparatus (BÜCHI Labortechnik AG, Flawil, Switzerland) and are uncorrected. ^1^H NMR spectra were obtained with a Bruker Ascend 500 MHz spectrometer (Bruker Italia S.r.l., Milano, Italy); δ values are in ppm, J values are in Hz. Compounds were dissolved in dimethylsulfoxide (DMSO). All exchangeable protons were confirmed by the addition of D_2_O. Mass spectra were recorded on an HPLC Alliance 2695 (Waters, Milford, MA, USA). Thin-layer chromatography (TLC) was carried out on pre-coated TLC plates with silica gel 60 F254 (Merk Life Science S.r.l., Milan, Italy). For column chromatography, silica gel 60 (Merck) or the Isolera Biotage four instrument (Biotage, Uppsala, Sweden) was used. Elemental analyses were determined on a Fisons Instruments Model EA 1108 CHNS-O model analyzer (Thermo Scientific, Waltham, MA, USA) and are within 0.4% of the theoretical values. The purity of the compounds is >99%, according to the elemental analysis data.

#### 2.1.2. Synthesis of Compounds

A synthesis of 9-ethyl-8-iodo-2-phenethoxy-9*H*-purin-6-amine (**5**): in a three-neck flask, in strong anhydrous conditions and under N_2_ atmosphere, diisopropylamine (freshly distilled over CaH_2_, 5 eq, 247 µL; 1.76 mmol), was solubilized in anhydrous THF (0.7 mL), and then butyllithium was added dropwise and the solution left at r.t. for 15 min. Then, the solution was cooled to −70 °C, and a solution of **3** (100 mg; 0.35 mmol) in anhydrous THF (1.1 mL) was added dropwise; the reaction mixture was left at −70 °C for 1 h, and then a solution of I_2_ (1.6 eq, 143 mg; 0.56 mmol) in anhydrous THF (2 mL) was added. The reaction was left for 2 h at −70 °C and warmed to r.t. in 30 min, in which the starting material was not completely consumed. The reaction was quenched by adding glacial CH_3_COOH (2 drops) and CH_3_OH (2 mL), and then volatiles were removed under vacuum and the crude mixture chromatographed on a normal column, and dry slurry was eluted with CHCl_3_–CH_3_OH 98:2. The compound **5** was obtained after crystallization from CH_3_OH as a white solid with 51% yield. M.p. = 196–198 °C (dec.). ^1^H-NMR (DMSO-*d_6_*) δ 1.28 (t, 3H, *J* = 7.0 Hz, CH_2_*CH_3_*), 2.97 (t, 2H, *J* = 6.9 Hz, CH_2_-Ph), 4.02 (t, 2H, *J* = 7.0 Hz, *CH_2_*CH_3_), 4.40 (t, 2H, *J* = 6.9 Hz, O*CH_2_*), 7.15–7.50 (m, 7H, H-Ph and NH_2_); ESI-MS positive mode *m*/*z*: 409.8 ([M + H]^+^), 431.7 ([M + Na]^+^); elemental analysis calculated for C_15_H_16_IN_5_O: C, 44.03; H, 3.94; I, 31.01; N, 17.11; found C, 44.20; H, 3.71; N, 17.18.

A synthesis of 8-bromo-9-ethyl-2-(4-methoxyphenethoxy)-9*H*-purin-6-amine (**10**): **9** (600 mg, 1.91 mmol) was solubilized in anhydrous DMF (13 mL), let to stir at r.t. under N_2_ atmosphere, and then NBS (1.5 eq, 2.87 mmol, 511 mg) was added. The reaction was complete after 10 min. Volatiles were removed under vacuum, the residue was chromatographed on a gravimetric column, and the dry slurry was eluted with CHCl_3_–CH_3_OH (98:2). A further purification is necessary by chromatography using a chromatotron eluting with cHex–AcOEt–CH_3_OH (70:28:2). The compound **10** was obtained as a pale-yellow solid after recrystallization from CH_3_OH with 48% yield. M. p. = 190–192 °C; ^1^H-NMR (DMSO-*d_6_*) δ 1.31 (t, *J* = 7.2 Hz, 3H, CH_2_-*CH_3_*), 2.95 (t, *J* = 7.4 Hz, 2H, CH_2_-Ph), 3.74 (s, 1H, O-CH_3_), 4.07 (q, *J* = 7.0 Hz, 2H, *CH_2_*-CH_3_), 4.37 (t, *J* = 7.0 Hz, 2H, O-CH_2_), 6.89 (d, *J* = 8.7 Hz, 2H, H-Ph), 7.24 (d, *J* = 8.6 Hz, 2H, H-Ph), 7.39 (bs, 2H, NH_2_); ESI-MS positive mode *m*/*z*: 391.8 ([M + H]^+^), 413.8 ([M + Na]^+^).

A synthesis of 2-(4-((6-amino-9-ethyl-9*H*-purin-2-yl)oxy)phenyl)ethan-1-ol (**13**): **8** (400 mg; 1.02 mmol) was solubilized in anhydrous CH_3_CN (4 mL) to the solution, finely ground NaOH (200 mg), and had 4-hydroxyphenethyl alcohol (1.00 g) added, and the reaction mixture was left at reflux for 24 h in an oil bath. Volatiles were removed under vacuum, the crude residue was chromatographed on a flash silica gel column, and dry slurry was eluted with CHCl_3_–CH_3_OH (98:2). Compound **13** was obtained as a white solid after recrystallization from CH_3_OH with 34% yield. M.p. = 228–230 °C; ^1^H-NMR (DMSO-*d*_6_) δ 1.36 (t, 3H, *J* = 7.2 Hz, CH_2_*CH_3_*), 2.73 (t, 2H, *J* = 6.9 Hz, CH_2_Ph), 3.64 (m, 2H, *CH_2_*OH), 4.05 (q, 2H, *J* = 7.2 Hz, *CH_2_*CH_3_), 4.67 (t, 1H, *J* = 6.5 Hz, OH), 7.03 (d, 2H, *J* = 8.6 Hz, H-Ph), 7.23 (d, 2H, *J* = 8.6 Hz, H-Ph), 7.32 (bs, 2H, NH_2_), 8.03 (s, 1H, H-8); ESI-MS positive mode *m*/*z*: 299.9 ([M + H]^+^), 322.0 ([M + Na]^+^); elemental analysis calculated for C_15_H_17_N_5_O_2_: C, 60.19; H, 5.72; N, 23.40; found 60.28; H, 5.65; N, 23.76.

### 2.2. Biological Evaluation In Vitro

#### 2.2.1. Binding Studies and Adenylyl Cyclase Activity at Human ARs

The radioligand binding experiments were carried out exactly as described previously [30]. For A_1_AR binding, 1 nM [^3^H]CCPA was used as a radioligand, whereas 30 and 10 nM [^3^H]NECA were used for A_2A_ and A_3_ ARs, respectively. Nonspecific binding was determined in the presence of 1 mM theophylline (A_1_AR) or 100 pM R-PIA (A_2A_ and A_3_ ARs). K_i_ values were calculated from competition curves by nonlinear curve fitting with the program SCTFIT [31].

CHO cells stably transfected with human ARs were grown adherently and maintained in Dulbecco’s Modified Eagles Medium with nutrient mixture F12 (DMEM/F12) without nucleosides, containing 10% fetal calf serum, penicillin (100 U/mL), streptomycin (100 μg/mL), L-glutamine (2 mM), and Geneticin (G-418, 0.2 mg/mL) at 37 °C in 5% CO_2_/95% air, as described earlier [30]. For radioligand binding studies and measurement of adenylyl cyclase activity, crude membrane fractions were prepared from fresh or frozen cells with two different protocols described earlier [30]. The determination of adenylyl cyclase activity followed the procedure described earlier [30]. IC_50_ values for the inhibition of adenylyl cyclase stimulated (in the case of A_2B_AR) with 5 μM NECA were calculated with the Hill equation and converted to *K*_i_ values with the Cheng and Prusoff equation [32]. The Hill slopes were near unity suggesting a competitive interaction of the antagonists tested.

#### 2.2.2. Functional Assays at the A_2A_AR

The performed GloSensorÏ cAMP assay is a non-radioactive method that offers a simple and powerful approach to monitor G-protein coupled receptor (GPCR) activity through change in the intracellular cAMP concentration [33].

Cell culture: CHO cells stably expressing human A_2A_AR were grown adherently and maintained in Dulbecco’s modified Eagle’s medium with nutrient mixture F12 (DMEM/F12 with phenol red), supplemented with 10% fetal bovine serum (FBS), 100 U mL^−1^ penicillin, 100 µg mL^−1^ streptomycin, 2.5 µg mL^−1^ amphotericin, 1 mM sodium pyruvate and 0.1 mg mL^−1^ geneticin (G418), at 37 °C and aerated with 5% CO_2_:95% O_2_. Cells were grown to approximately 70–80% confluence, and transient transfection with a plasmid encoding the biosensor was performed.

GloSensorÏ cAMP assay: Cells were harvested in CO_2_-independent medium and were counted in a Neubauer chamber. The desired number of cells was incubated in equilibration medium containing a 3% *v*/*v* GloSensor^TM^ cAMP reagent stock solution, 10% FBS, and 87% CO_2_ independent medium. After 2 h of incubation, the cells were dispensed in the wells of a 384-well plate and, when a steady-state basal signal was obtained, the NECA referent agonist or the understudy compounds, at different concentrations, were added. The new synthesized compounds did not produce stimulation of cAMP, so they were evaluated as antagonists. The antagonist profile was evaluated by assessing their ability to counteract an agonist-induced increase in cAMP accumulation. The cells were incubated in the reaction medium (10 min at room temperature) with different understudy molecule concentrations and then treated with NECA. After 10 min, various luminescence reads were performed at different incubation times.

Statistical analysis: Responses were expressed as a percentage of the maximal relative luminescence units (RLU). Concentration–response curves were fitted by a nonlinear regression with the Prism 4.0 program (GraphPAD Software, San Diego, CA, USA). To quantify the NECA agonist potency, the EC_50_ value was calculated. The EC_50_ value is the concentration of agonists required to produce 50% of the maximum effect. To evaluate the antagonist profile, the IC_50_ values were determined. The IC_50_ value is the concentration of antagonists that produces a 50% inhibition of the agonist effect. Each concentration was tested three times in triplicate, and the values are given as the mean ± standard error.

### 2.3. Biological Evaluation In Vivo

#### 2.3.1. Animals

Male Sprague Dawley rats (200–300 g, Charles River, Calco, Italy) were housed in groups of 4–6 in standard polycarbonate cages with sawdust bedding and maintained on a 12 h light/dark cycle (lights on at 8:00 am). Food and water were freely available. All experiments were conducted in accordance with the guidelines for animal experimentation of the EU directives (2010/63/EU; L.276; 22 September 2010) and approved by the Committee on Animal Experimentation (CESA; protocol code 29456, 21 March 2012) of the University of Cagliari. Experiments were designed to minimize animal discomfort and to reduce the number of animals used.

#### 2.3.2. Catalepsy

Catalepsy was estimated using the vertical grid test. The test was carried out by placing a rat with the four paws on a wire grid (43–25 cm) at an angle of about 70° in respect to the bench surface. Catalepsy was determined by measuring the time in which the rat maintained a given position. The test was terminated when the rat moved one paw or when 90 s had elapsed from the placement of the rat on the grid. Catalepsy assessments were repeated every 10–15 min intervals. At each test time, rats that did not assume the given position on the grid after three attempts were classified as 0 s latency. A_2A_AR antagonists were injected 90 min after haloperidol administration to evaluate their effects on deeply cataleptic rats [18].

#### 2.3.3. Tacrine-Induced Tremulous Jaw Movements

Rats were divided into two groups and treated with vehicle or **4** (5 mg/kg. i.p.). Tacrine (2.5 mg/kg i.p.) was administered 20 min after vehicle or **4**, and the number of TJMs and bursts of TJMs (i.e., episodes of consecutive TJMs) were measured for 60 min. TJMs were defined as vertical deflections of the lower jaw not directed at a particular stimulus [23]. Yawns, tongue protrusions, and stereotypies, such as grooming, were not scored.

#### 2.3.4. 6-OHDA-Lesion

Rats (275–300 g) were anesthetized and placed on a David Kopf stereotaxic apparatus (Tujunga, CA, USA) and infused, using a stainless steel cannula, into the left medial forebrain bundle [coordinates A = −2.2, L = +1.5 from bregma, V = −7.9 from the dura, according to the atlas of Pellegrino et al. (1979) [34]] with 6-OHDA (8 μg/4 μL of saline containing 0.05% ascorbic acid). Rats were pretreated with desipramine (10 mg/kg i.p.) to prevent damage to noradrenergic neurons [18,35].

#### 2.3.5. Assessment of Rotational Behavior

Rotational behavior was assessed in hemispherical bowls (50 cm diameter), with sawdust on the floor, in which each rat was connected to an automated rotameter system (Panlab s.l., Barcelona, Spain) capable of detecting the number of full (360°) rotations in both directions (ipsilateral and contralateral to the lesioned hemisphere) [35]. Rats were placed in the bowls 30 min before drug administration to acclimatize and extinguish any spontaneous rotational behavior, and both contralateral and homolateral rotations were measured in 10 min blocks for 120 min after drug injection. The total number of contralateral and homolateral rotations (mean ± SEM) in a 2 h testing period were calculated.

Potentiation of L-dopa-induced rotational behavior: two weeks after the unilateral 6-OHDA lesion, rats were screened based on their contralateral rotations in response to L-dopa (50 mg/kg i.p.) + benserazide (30 mg/ kg i.p.). Rats not showing at least 300 contralateral rotations during the 2 h testing period were eliminated from the study. Three days later, rats were administered with a subthreshold dose of L-dopa (3 mg/kg i.p.) + benserazide (6 mg/kg i.p.) in combination with the vehicle or with a dose of 5 mg/kg i.p of **1**, **4**, or **5**. Compounds were administered simultaneously with L-dopa. Benserazide was always injected 30 min before L-dopa.

#### 2.3.6. Drugs

Compounds **1**, **4**, and **5** were dissolved by adding DMSO, polyethylene glycol (PEG 400), and water in a ratio of (50:350:600 μL) and vortexing vigorously; the clear solution was injected in a volume of 0.3 mL i.p. per 100 g body weight. 6-OHDA–HCl, desipramine, benserazide, and L-dopa were purchased from Sigma-Aldrich Co. (St. Louis, MO, USA). Haloperidol was purchased from a commercial source (Serenase, Lusofarmaco, Italy), diluted in distilled water, and administered s.c. The drugs administered parenterally were dissolved in saline and injected in a volume of 0.3 mL i.p. per 100 g body weight or a volume of 0.1 mL s.c. × 100 g body weight.

The dose of 5 mg/kg used for the derivatives **1**, **4**, and **5** was chosen based on preliminary studies showing that 1 and 3 mg/kg of **1**, **4**, and **5** had low efficacy on catalepsy, whereas 5 mg/kg was fully effective, similar to other A_2A_AR antagonists with the same affinity [18]. In order to reduce the number of animals used, the dose of 5 mg/kg was used in every *in vivo* experiment.

#### 2.3.7. Data Analysis and Statistics

For catalepsy evaluation, the mean and S.E.M. of the seconds of immobility during each test section were calculated. Significant differences between groups were evaluated by one-way analysis of variance (ANOVA) followed by a Newman–Keuls post hoc test.

In the rotational behavior experiments, the mean and S.E.M. of the total number of contralateral rotations were calculated. Significant differences between groups were evaluated by one-way ANOVA followed by a Newman–Keuls post hoc test.

In the tacrine-induced TJM tests, the mean and S.E.M of the number of TJMs and bursts were calculated. Significant differences between groups were evaluated by Student’s *t*-test. 

## 3. Results and Discussion

### 3.1. Chemistry

Synthesis of 8-substituted 2-phenethoxyadenine derivatives **4**–**7** were accomplished starting from **1** to **3** [19]. The 8-bromoadenine derivative **1** was reacted with ethanol in the presence of sodium hydroxide, under heating, to obtain the 8-ethoxy-9-ethyl-2-phenethoxyadenine (**4**). Treatment of **1** with 2-(tributylstannyl)thiophene or furane under Stille reaction conditions using triphenylphosphine palladium dichloride in anhydrous tetrahydrofurane (THF) furnished the 8-heteroaryl derivatives **2** and **6**. The reduction of **2** to the 8-tetrahydrofuryladenine derivative **7** was obtained using hydrogen atmosphere and palladium oxide as a catalyst in isopropanol and acidic conditions at 65 °C (Scheme 1).

For the synthesis of the new 8-iodo derivative **5**, the 9-ethyl-2-phenethoxyadenine (**3**) was used as the starting material. The introduction of the iodine atom at the 8-position was obtained by a reaction of the adenine derivative **3** with freshly prepared lithium diisopropyl amide in strictly anhydrous conditions. The 8-lithium intermediate was then reacted with elementary iodine to obtain **5** after purification on silica gel chromatography.

The synthesis of adenine derivatives **9**–**11**, bearing a methoxy group at the phenyl ring of the 2-chain, was accomplished starting from the 2-chloro-9-ethyladenine (**8**) [36] (Scheme 2). Hence, **8** was treated with 4-methoxyphenyl alcohol and sodium hydroxide in CH_3_CN, heating at 85 °C, to obtain the 9-ethyl-2-(4-methoxyphenyl)ethoxyadenine (**9**). The latter compound was reacted with *N*-bromosuccinimide (NBS) at r.t. in anhydrous conditions using dimethylformamide (DMF) as a solvent. The reaction was very fast, and **10** was obtained with 48% yield. Treatment of **10** with tributylstannyl furane under Stille coupling reaction conditions gave the 8-furyl derivative **11**. With the aim at obtaining the phenol chain in 2-position (compound **12**), removal of the methyl group from **10** was attempted in hydrolytic conditions with an aqueous solution of HBr and heating at 100 °C, using the same procedure recently reported for similar compounds [36]. The reaction failed, leading to the removal of the whole chain from the 2-position of the purine ring. Alternatively, **8** was reacted with the 4-hydroxyphenethyl alcohol in similar conditions for the synthesis of **9**. Unfortunately, the reaction led to the formation of the new compound **13**, in which the phenolic hydroxyl group reacted in position 2 of the purine ring.

### 3.2. Biological Activity In Vitro

#### 3.2.1. Binding Studies

Binding studies, performed at human-recombinant ARs stably transfected in Chinese hamster ovary (CHO) cells, allowed us to evaluate the affinity of compounds **4**–**7**, **9**–**11**, and **13** at A_1_, A_2A_, and A_3_ ARs. [^3^H]CCPA (2-chloro-*N*^6^-cyclopentylAdo) and [^3^H]NECA (5′-*N*-ethylcarboxamidoAdo) were used as specific radioligands at A_1_ARand A_2A_ / A_3_ ARs, respectively [30]. Furthermore, the ability of the same compounds to inhibit NECA-stimulated adenylyl cyclase activity was evaluated at the A_2B_AR subtype [30]. The results of the binding studies (A_1_, A_2A_, and A_3_ ARs) and functional data (A_2B_AR) are reported in Table 1 as *K*_i_ or IC_50_ values in nM, together with the data on the reference compounds **1**–**3**.

Compounds **1** and **2,** bearing a phenethoxy chain combined with a bromine atom or a furyl ring at the 8-position of 9-ethyladenine, are endowed with a very high affinity for the A_2A_AR (**1**; *K*_i_A_2A_AR = 1.7 nM and **2**; *K*_i_A_2A_AR = 2.2 nM) and a moderate selectivity for the same receptor subtype (**1**; selectivity A_1_/A_2A_ = 14 and A_3_/A_2A_ = 641 and **2**; selectivity A_1_/A_2A_ = 2.6 and A_3_/A_2A_ = 7.3). It is worthwhile to note that the presence in these derivatives of a substituent at the 8-position favors the interaction with all of the ARs. In fact, the corresponding 8-unsubstituted derivative **3** exhibited a *K*_i_A_2A_AR = 120 nM and lower affinity also at A_1_ and A_3_ receptor subtypes. Its activity at the A_2B_AR is lower as well, in respect to the 8-substitued analogs **1** and **2**.

As for compounds **1**–**3**, the here-reported 2,8-disubstituted 9-ethyladenine derivatives **4**–**7**, **9**–**11**, and **13** exhibited, in general, high affinity for the A_2A_AR, and are A_2A_ selective. Replacement of the 8-bromo and 8-furyl substituents of **1** and **2** with an ethoxy chain led to a derivative which maintains an affinity in the low nanomolar range at the A_2A_AR (**4**; *K*_i_A_2A_ = 3.7 nM), together with a good degree of selectivity versus the other subtypes (selectivity A_1_/A_2A_ = 23 and A_3_/A_2A_ = 97).

The 8-iodo derivative **5** resulted in the compound endowed with the highest affinity for the A_2A_AR of the whole series. In fact, it exhibited a *K*_i_A_2A_ = 1.2 nM combined with a good A_2A_ selectivity (selectivity A_1_/A_2A_ = 21 and A_3_/A_2A_ = 408).

The isosteric substitution of the furyl ring with a thiophene led to a threefold decrease in the A_2A_AR affinity in respect to **2** and comparable selectivity (**6**; *K*_i_A_2A_ = 7.7 nM, selectivity A_1_/A_2A_ = 2.7 and A_3_/A_2A_ = 3); reduction in the **2** furyl ring to the tetrahydrofuryl moiety led to a further decrease in the A_2A_AR affinity (**7**; *K*_i_A_2A_ = 81 nM).

The introduction of a methoxy group in para-position of the 2-phenyl ring led to a decrease in affinity at all ARs both in the presence of the 8-substitution (compounds **10** and **11**) and in the case of the 8-unsubstituted derivative **9**. As for the 8-unsubstituted derivative **3**, the presence of the 8-bromo or 8-furyl substituents enhanced the binding ability, leading to compounds endowed with good A_2A_AR affinity (**9**; *K*_i_A_2A_ = 655 nM, **10**; *K*_i_A_2A_ = 28 nM, and **11**; *K*_i_A_2A_ = 7.1 nM).

Finally, the 8-unsubstituted analog **13** showed µM affinity at A_1_ and A_2A_ AR subtypes and was not able to bind the other receptors at concentrations up to 100 µM.

Functional experiments demonstrated that most of the compounds antagonized the A_2B_AR at µM or sub-µM concentrations.

#### 3.2.2. Functional Experiments

On the base of the affinity and selectivity results, compounds **1, 4**, and **5** were selected for functional studies at the A_2A_AR in order to verify their antagonistic behavior through the evaluation of the inhibition of cAMP production. Before that, they were tested alone to exclude their agonist behavior. In fact, when tested alone, no increase in cAMP production was detected (Figure S1 in Supplementary Materials).

Hence, the inhibitory effects of **1**, **4**, and **5** on the NECA-induced cAMP production in CHO cells stably expressing human A_2A_ARs was evaluated using GloSensor^TM^ cAMP assays [37]. The results of these experiments are reported in Table 2.

Compounds **1**, **4**, and **5** inhibited NECA-induced cAMP production, therefore behaving as A_2A_AR antagonists. In particular, they exhibited IC_50_ values in the high nM range (**1**: IC_50_ = 108 nM; **4**: IC_50_ = 278 nM; and **5**: IC_50_ = 114 nM). It is worthwhile to note that the IC_50_ values were perfectly in agreement with the *K*_i_s found in binding studies.

### 3.3. Biological Activities in In Vivo Models of Parkinson’s Disease

#### 3.3.1. Effect of A_2A_AR Antagonists on Catalepsy

Compounds **1**, **4**, and **5** did not modify spontaneous motility in rats when tested at 5 mg/kg i.p., whereas they caused hypermotility at higher doses of 10 and 15 mg/kg similar to proven A_2A_AR antagonists [18,24]. The choice of the 5 mg/kg i.p. dose was decided on the base of preliminary studies, which showed that this dose was fully effective while the lower doses of 1 mg/kg of **1**, **4**, and **5** had low efficacy on catalepsy [18].

After 40 min of haloperidol administration (0.2 mg/kg s.c.), a significant catalepsy was induced in rats, which reached its maximum at 60–70 min [18,24]. Therefore, the administration of the A_2A_AR antagonists was made 90 min after haloperidol to evaluate their effects on deeply cataleptic rats.

**Figure 2:**
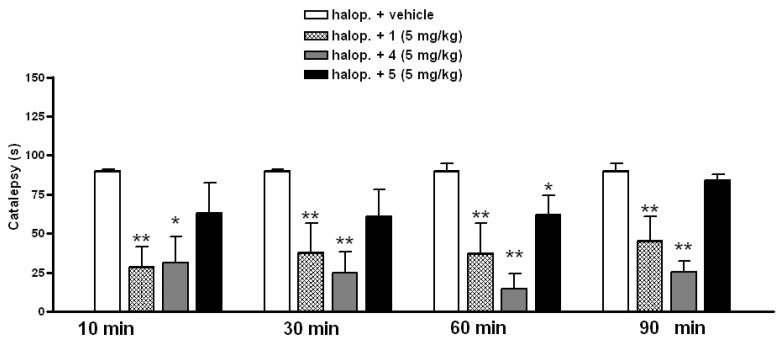
Effect of A_2A_AR antagonists on catalepsy induced by haloperidol. Effect of **1** (5 mg/kg i.p.; n = 5), **4** (5 mg/kg i.p.; n = 6), or **5** (5 mg/kg i.p.; n = 5) on catalepsy induced by haloperidol (0.2 mg/kg s.c.; n = 7) in rats, 10, 30, 60, and 90 min after drug administration. Results are mean ± S.E.M. of the intensity of catalepsy, measured as the time spent in cataleptic posture by each rat in the test section. Statistical significance was determined by one-way ANOVA followed by Newman–Keuls’ post hoc test. ** *p* < 0.0001, * *p* < 0.05 versus haloperidol + vehicle.

At a dose of 5 mg/kg i.p., **1**, **4**, and **5** significantly antagonized catalepsy induced by 0.2 mg/kg of haloperidol during the 90 min testing period (Figure 2). Specifically, the anticataleptic effect of **5** was narrowed to 60 min, whereas **1** and **4** elicited an anticataleptic effect of longer duration (between 10–90 min). Specifically, the anticataleptic efficacy of **4** had a longer duration and a strong intensity, as shown in Figure 2;, indeed, rats did not return to a cataleptic state even after the 90 min of testing-period.

#### 3.3.2. Potentiation of L-Dopa-Induced Rotational Behavior

**Figure 3:**
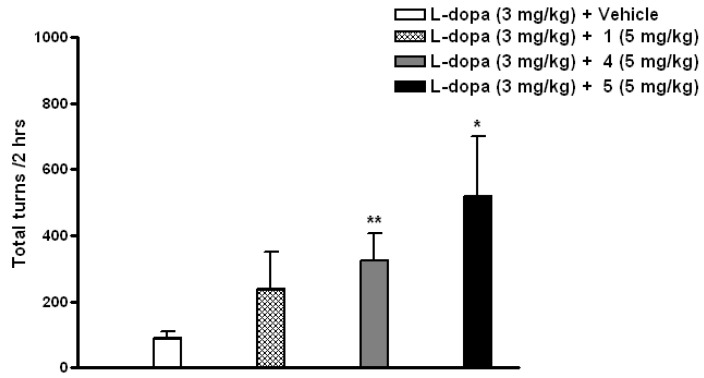
Potentiation of L-dopa-induced contralateral turning by A_2A_AR antagonists. Effect of administration of L-dopa (3 mg/kg i.p.) + vehicle (n = 8), L-dopa (3 mg/kg i.p.) + **1** (5 mg/kg i.p.; n = 5), L-dopa (3 mg/kg i.p.) + **4** (5 mg/kg i.p.; n = 7), L-dopa (3 mg/kg i.p.) + **5** (5 mg/kg i.p.; n = 5). Ordinate indicates the total number of turns measured in 2 h; values represent contralateral rotations, respectively. Results are mean ± S.E.M. of total turns. Statistical significance was determined by one-way ANOVA followed by Newman–Keuls’ post hoc test. ** *p* < 0.0001, * *p* < 0.05 versus L-dopa alone.

Acute administration of **1** (5 mg/kg i.p.) did not increase the number of contralateral rotations induced in 6-OHDA-lesioned rats by a subthreshold dose of L-dopa (3 mg/kg i.p.; Figure 3), whereas the acute administration of either compounds **4** or **5** significantly increased the number of contralateral rotations induced by L-dopa (3 mg/kg) in 6-OHDA-lesioned rats (Figure 3). Contralateral turning, shown by 6-OHDA-lesioned rats in comparison with those receiving L-dopa, is presented in Figure S2 in the Supplementary Materials.

#### 3.3.3. Effect of 4 on Tacrine-Induced Tremulous Jaw Movements

**Figure 4:**
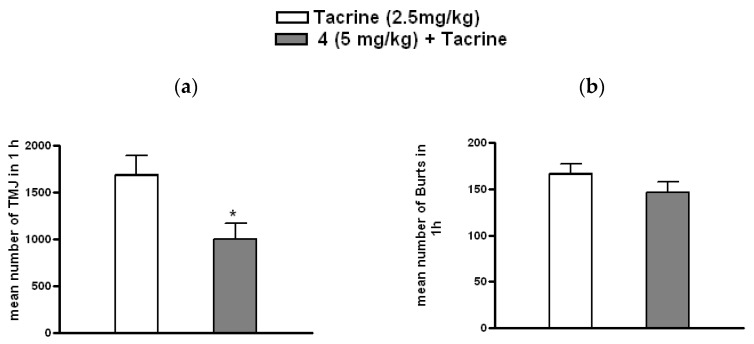
Effect of **4** on tacrine-induced parkinsonian-like tremors. Effect of **4** (5 mg/kg i.p.) pre-treatment on tremulous jaw movements and tremor bursts induced in rats by tacrine (2.5 mg/kg i.p.). Results are mean ± SEM of tremulous jaw movements (**a**) and mean ± SEM of tremor bursts (**b**) recorded for 60 min after tacrine administration. Compound **4** significantly reduced tremulous jaw movements, but not tremor bursts, as compared to the vehicle. Statistical significance was determined by Student’s *t*-test. * *p* < 0.05 versus vehicle; (n = 4–6).

Since the A_2A_AR antagonist **4** was the most effective compound in the tests of catalepsy and the potentiation of L-dopa-induced rotational behavior, it was further evaluated for its effects on tacrine-induced TJMs. The results reveal that pretreatment with **4** (5 mg/kg) significantly reduced the number of TJMs induced by acute administration of tacrine (2.5 mg/kg) (*p* < 0.05 vs. vehicle) (Figure 4a). Nevertheless, pretreatment with **4** (5 mg/kg) did not affect the number of tremor bursts induced by tacrine (*p* < 0.05 vs. vehicle) (Figure 4b).

Consistent with previous studies, the results in the *in vivo* model of PD of compounds **1**, **4,** and **5**, demonstrated that binding to A_2A_ARs resulted in functional antagonistic actions on this receptor. All three agents reversed haloperidol-induced catalepsy, showing a pharmacological profile similar to proven A_2A_AR antagonists [18,24]. The evaluation of the anticataleptic effect revealed its shorter duration for compound **5** and longer duration and stronger anticataleptic efficacy for derivative **4** compared to compounds **1** and **5**. The effects of compounds **1**, **4,** and **5** were further investigated using the rotational 6-OHDA-model, where, similarly to other selective A_2A_AR, the **4** and **5** compounds potentiated the contralateral rotations induced by a subthreshold dose of L-dopa [18,24].

As described above, derivative **4**, showing the highest antiparkinsonian efficacy in both the catalepsy and 6-OHDA-model of PD, was further investigated in the tacrine model of parkinsonian-like tremors [23,24,25,29]. Consistent with preclinical and clinical studies in which other A_2A_AR antagonists showed anti-tremorigenic effects, derivative **4** reduced the intensity of TJMs induced by tacrine, showing a clear action of this compound on parkinsonian-like tremors [23,24,28,29,38,39,40].

Overall, the *in vivo* experiments in the rodent model of PD with compounds **1**, **4**, and **5** demonstrated that derivative **1**, although it antagonized catalepsy induced by haloperidol, was not effective in the contralateral rotational behavior test. The motives for this discrepancy are unknown; nevertheless, since after 6-OHDA lesion dopamine D_2_ receptors become supersensitive (increased number), it is possible that the antagonism of A_2A_AR is less effective in potentiating a higher number of D_2_ receptors [41,42,43]. In addition, since the potentiation of contralateral rotations induced by L-dopa involves the integrated stimulation of both dopamine D_1_ and D_2_ receptors that have a different localization in basal ganglia efferent pathways, A_2A_AR antagonists might have a different effect on the two pathways [7,18,24,43,44,45].

Finally, compound **4** resulted in the derivative with the most complete antiparkinsonian profile; indeed, this compound showed a strong anticataleptic effect with a longer duration that might be related to its longer half-life, a good efficacy in potentiating the contralateral rotations induced by L-dopa and in reducing TJMs in the tacrine model, suggesting its efficacy against parkinsonian symptoms such as akinesia and tremor.

## 4. Conclusions

A series of 2,8-disubstituted 9-ethyladenine derivatives were synthesized and tested in binding and functional studies at ARs, stably transfected in CHO cells. All compounds were able to bind A_1_, A_2A_, and A_3_ ARs with *K*_i_ values ranging from low nM to microM concentrations, resulting in being selective for the A_2A_AR subtype. Their ability to inhibit NECA-stimulated AC activity at A_2B_ARs was moderate. Compounds **1**, **4**, and **5**, endowed with the best combination of affinity and selectivity at A_2A_ARs, were evaluated in functional studies, which confirmed their A_2A_AR antagonist behavior. Hence, they were evaluated in *in vivo* rat models of PD and were found to be able to revert haloperidol-induced catalepsy and to potentiate L-dopa-induced contralateral rotations in the 6-OHDA model. The most efficacious compound, **4**, was further investigated on tacrine-induced TJMs, where it reduced the intensity of tremulous jaw movements, showing efficacy in reducing parkinsonian tremors. All of these results demonstrate that 8-ethoxy-2-phenethoxy-9-ethyladenine (**4**) is a good candidate to be investigated as a new antiparkinsonian drug.

## Figures and Tables

**Table 1:**
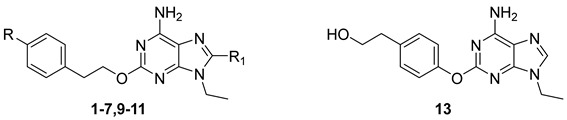
Radioligand binding affinities of compounds **1**–**7**, **9**–**11**, and **13** at A_1_, A_2A_, and A_3_ ARs (*K*_i_, nM) and functional studies activity at the A_2B_AR (IC_50_, nM) subtype at human ARs stably transfected in CHO cells.

Cpd.	R	R_1_	*K*_i_ or IC_50_ (nM)				Selectivity	
			A_1_ ^a^	A_2A_ ^b^	A_2B_ ^c^	A_3_ ^d^	A_1_/A_2A_	A_3_/A_2A_
**1** [19]	H	Br	23 (23–24)	1.7 (1.4–2.2)	569 (440–734)	1090 (685–1720)	14	641
**2** [20]	H	2-Furyl	5.8 (4.0–8.4)	2.2 (2.1–2.3)	521 (467–580)	16 (11–22)	2.6	7.3
**3** [19]	H	H	170 (130–230)	120 (70–220)	45,800 (29,800–70,500)	7150 (2950–17,300)	1.4	59
**4**	H	O-CH_2_CH_3_	86 (70–106)	3.7 (3.5–3.8)	6510 (6200–6830)	359 (290–444)	23	97
**5**	H	I	25 (21–31)	1.2 (1.1–1.4)	719 (464–1120)	489 (323–741)	21	408
**6**	H	2-Thiophenyl	21 (17–25)	7.7 (7.1–8.2)	4990 (4060–6140)	27 (21–36)	2.7	3.5
**7**	H	2-THF	509 (426–608)	81 (49–136)	38,900 (32,956–44,660)	1450 (1310–1600)	6.3	18
**9**	OMe	H	1986 (1470–2680)	655 (526–816)	63,700 (40,348–121,220)	12,300 (11,800–12,800)	3.0	19
**10**	OMe	Br	197 (152–253)	28 (21–37)	9190 (8800–9600)	4060 (3390–4850)	7.1	146
**11**	OMe	2-Furyl	39 (33–47)	7.1 (5.6–9.0)	>100 µM	73 (53–100)	5.5	10
**13**			22,900 (17,300–30,200)	7080 (5300–9440)	>100 µM	>100 µM	3.2	14

^a^ Displacement of specific [^3^H]-CCPA binding at human A_1_AR expressed in CHO cells, (n = 3–6). ^b^ Displacement of specific [^3^H]-NECA binding at human A_2A_AR expressed in CHO cells. ^c^ IC_50_ values of the inhibition of NECA-stimulated AC activity in CHO cells expressing human A_2B_AR. ^d^ Displacement of specific [^3^H]-NECA binding at human A_3_AR expressed in CHO cells. Data are expressed as geometric means with 95% confidence limits.

**Table 2:** Inhibitory effect of **1**, **4**, and **5** on the NECA-induced cAMP production in CHO cells expressing hA_2A_ARs. Binding data are reported for comparison.

Compd.	IC_50_ [nM] ^1^	*K*_i_ [nM]
**1**	108 ± 23	1.7
**4**	278 ± 49	3.7
**5**	114 ± 25	1.2

^1^ Concentration of antagonists that produces 50% inhibition of the agonist effect. Each concentration was tested three times in triplicate, and values are given as the mean ± standard error.

## Data Availability

The original contributions presented in this study are included in the article/Supplementary Materials. Further inquiries can be directed to the corresponding author.

## Supplementary Materials

The following supporting information can be downloaded at: https://www.mdpi.com/article/10.3390/cells14050338/s1, Figure S1: cAMP production induced by compounds **1**, **4**, **5** in A_2A_AR transfected CHO cells in comparison with NECA; Figure S2: Contralateral rotation induced by L-dopa.
