# Supplementary material for: A2A Adenosine Receptor Antagonists and Their Efficacy in Rat Models of Parkinson’s Disease
Source: Cells. 2025 Feb 26;14(5):338. doi: 10.3390/cells14050338 (PMC11898488; doi:10.3390/cells14050338)
Supplement: Supplementary file 1 [file cells-14-00338-s001.zip › cells-3428950-supplementary.pdf]

A_{2A} adenosine receptor antagonists and their efficacy in rat models of Parkinson's disease

Andrea Spinaci, Michela Buccioni, Diego Dal Ben, Beatrice Francucci, Karl-Norbert Klotz, Gabriella Marucci, Nicola Simola, Micaela Morelli, Annalisa Pinna, Rosaria Volpini, Catia Lambertucci

Supplementary Materials

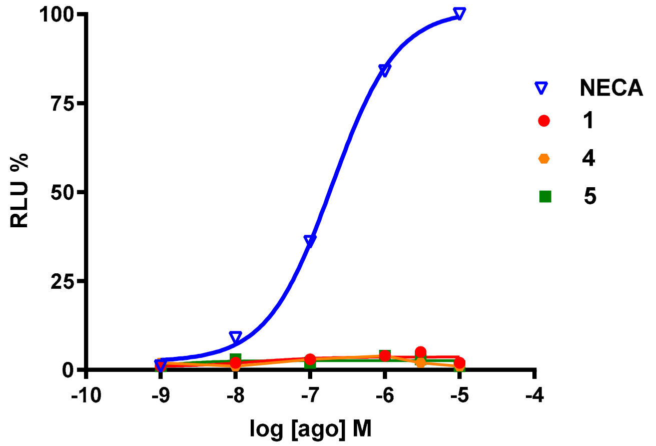

Figure S1. cAMP production induced by compounds 1, 4, 5 in A_{2A}AR transfected CHO cells in comparison with NECA. The cAMP production is revealed by luminescence produced after activation of AC as signal transduction of the receptor stimulation. The luminescence is measured as relative luminescence unit % (RLU%). Differently from NECA, compounds **1**, **4**, **5** are not able to stimulate cAMP production when tested alone.

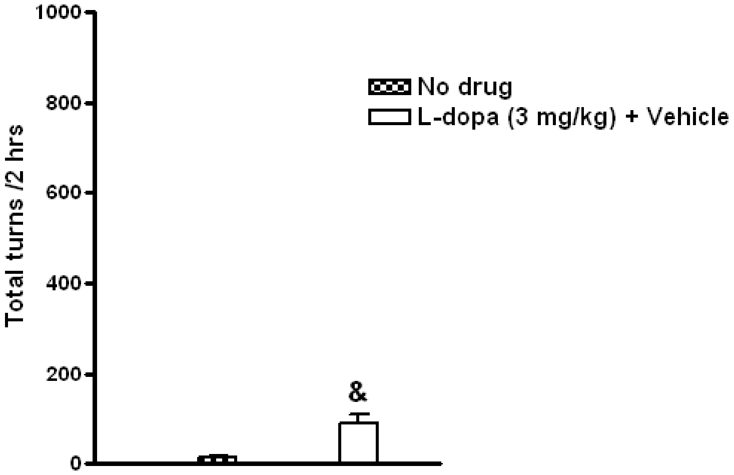

Figure S2. Contralateral rotation induced by L-dopa. Effect of no drug administration (n = 8) and administration of L-dopa (3 mg/kg i.p.) + vehicle (n = 8). Ordinate indicates the total number of turns measured in 2 hours; values represent contralateral rotations. Results are mean ± S.E.M. of total turns. Statistical significance was determined by one-way ANOVA followed by Newman–Keuls post hoc test. & $p < 0.05$ versus L-dopa alone. L-dopa induces contralateral turning rotation; when L-dopa is not administered, the animals do not show contralateral turning rotation.
